# Multifunctional Microspheres Based on D-Mannose and Resveratrol for Ciprofloxacin Release

**DOI:** 10.3390/ma15207293

**Published:** 2022-10-18

**Authors:** Roberta Cassano, Federica Curcio, Debora Procopio, Marco Fiorillo, Sonia Trombino

**Affiliations:** Department of Pharmacy, Health and Nutritional Sciences, Università della Calabria, Arcavacata di Rende, 87036 Cosenza, Italy

**Keywords:** ciprofloxacin, D-mannose, resveratrol, microspheres

## Abstract

This article describes the preparation, characterization, and performance evaluation of functional microspheres useful for the release of ciprofloxacin. The particles were obtained using D-mannose, a natural aldohexose sugar, and resveratrol, a powerful antioxidant. In particular, the above compounds were initially converted into D-mannose carboxylate and resveratrol methacrylate and, therefore, subjected to an esterification reaction. The resulting product was used for the preparation of the microspheres which were characterized by light scattering, FT-IR spectrophotometry and scanning electron microscopy (SEM). Subsequently, their degree of bloating was evaluated at pH 1.2 to simulate the pH of the stomach, at pH 6.8 and pH 7.4 to mimic the intestinal environment. The antibiotic ciprofloxacin was then loaded into the microspheres, with an encapsulation efficiency of 100%. The cumulative amount of drug released was 55% at pH 6.8 and 99% at pH 7.4. The tests conducted to evaluate the antibacterial activity demonstrated the ability of the microspheres obtained to inhibit the growth of *Escherichia coli*. The antioxidant efficacy, due to the presence of resveratrol in their structure, was confirmed using rat liver microsomal membranes. The results obtained have highlighted how the microspheres based on D-mannose and resveratrol can be considered promising multifunctional vectors useful in the treatment of intestinal and urinary infections.

## 1. Introduction

Ciprofloxacin is a second-generation fluoroquinolone antibiotic used primarily to treat urinary tract infections [1] caused by *E. coli* a bacterium commonly found in the gut but which, in some circumstances, can spread and colonize the circulatory system, central nervous system and the urinary tract [2]. In the latter case, the main complication to be resolved is due to the ability of *E. coli* to form biofilms with consequent persistence of this bacterium in the epithelium of the bladder [3]. Biofilms, bacterial communities embedded in the exopolysaccharide matrix, are dangerous as they protect bacteria from antibiotics action and make them a thousand times more resistant [4,5]. Therefore, the release of ciprofloxacin in the intestine is essential to avoid bacterial proliferation and, consequently, the onset of urinary tract infections. In addition to ciprofloxacin, alternative non-antibiotic methods such as probiotics, cranberry juice and D-mannose are being considered in recent years to treat urinary infections. The latter is a natural aldohexose sugar which is metabolized and excreted in the urine and acts by inhibiting the adhesion of bacteria to the urothelium. This bacterium, in fact, has filamentous appendages on its surface, called fimbriae, at the end of which there are adhesins (FimH) with high affinity for the glucose residues of cell surface glycoproteins that give *E. coli* the ability to adhere to cells bladder epithelial and colonize surrounding tissues.

D-mannose has a very similar structure to the binding site of cellular glycoprotein receptors, acting as a competitive inhibitor of bacterial adhesion and resulting as a good [6,7,8,9,10,11,12]. Resveratrol is a natural substance that has an antibacterial effect resolving biofilm. In fact, resveratrol is a natural antioxidant that is very attractive for its potential health benefits, including anticancer and anti-aging properties. In relation to its antimicrobial properties, it appears to be effective alone and in combination with conventional antibiotics [13,14,15,16,17].

**Figure 1:**
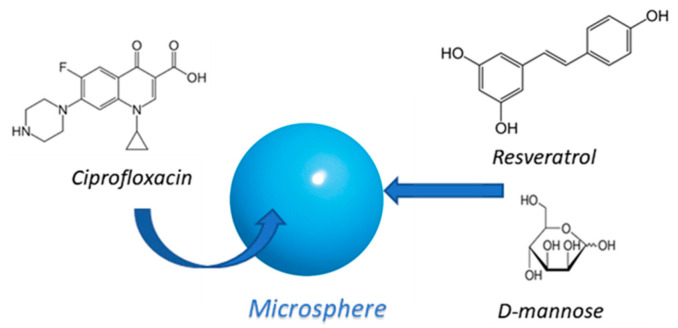
D-mannose and resveratrol-based microsphere containing ciprofloxacin.

Based on the beneficial properties of the substances described above, the idea was to design multifunctional microspheres for the intestinal release of ciprofloxacin, based on D-mannose and resveratrol in their matrix (Figure 1).

The microspheres were obtained by using reverse-phase emulsion polymerization. In particular, the derivative based on D-mannose carboxylate and resveratrol methacrylate, was reacted with the comonomer N,N′-ethylene bis-acrylamide (EBA) used as crosslinking agent. The particles were subsequently characterized by light scattering, FT-IR spectrophotometry and scanning electron microscopy (SEM). Furthermore, their degree of swelling was assessed at different pH values and at defined time points. The encapsulation efficiency of ciprofloxacin within the microspheres and the cumulative amount of drug released were then measured. Studies were also conducted to evaluate both the antibacterial activity, against *Escherichia coli*, and the antioxidant efficacy, due to the presence of resveratrol in the matrix of the microspheres.

## 2. Materials and Methods

### 2.1. Materials

The solvents used: acetone, ethanol, methanol, n-hexane, chloroform, 2-propanol, and ethyl ether were purchased from Carlo Erba Reagenti (Milan, Italy). Chloroform was purified by standard procedures. Resveratrol or 3,5,4’-trihydroxy-trans-stilbene (MM = 22,825 g/mol), D-Mannose (MM = 180,156 g/mol), ciprofloxacin (MM = 331,346), methacrylic acid (MAA), 85% formic acid (FA), 85% phosphoric acid (AP), sodium nitrite (SN), dicyclohexylcarbodimide (DCC), dimethylaminopyridine (DMAP), dimethyl sulfoxide (DMSO), potassium chloride (KCl), hydrochloric acid (HCl), sodium phosphate (Na_3_PO_4_), citric acid, disodium hydrogen phosphate (Na_2_HPO_4_), sorbitan monooleate (Span 80) and polyoxyethylene sorbitan monooleate (Tween 80), N,N′-ethylene bis-acrylamide (EBA) were purchased from Sigma-Aldrich (Sigma Chemical CO, St. Louis, MO, USA).

### 2.2. Instrumentation

FT-IR spectra were recorded on a Jasco 4200 spectrophotometer using potassium bromide (KBr) sheets provided by Sigma-Aldrich. The ¹H-NMR spectra were processed by means of a Bruker VM 30 spectrometer; the chemical shifts are expressed in ∂ and refer to the solvent. UV-Vis spectra were recorded by JASCO V-530 UV/Vis (Thermo Fisher Scientific, Monza, Italy) spectrophotometer using 1 cm thick quartz cells. The dimensional analysis of the microparticles was carried out both by light scattering, using a Brookhaven 90 Plus Particle Size Analyzer (Holtsville, NY, USA) and by Transmission Electron Microscopy (TEM) using a ZEISS EM 900 (Oberkochen, Germany) electron microscope at an accelerating voltage of 80 kV.

### 2.3. Animals

The animal study protocol was approved by the Italian Ministry of Health (Rome, Italy); (protocol code 700A2N.6TI, date of approval: March 2018). The animal procedures were conducted according to guidelines approved by the University Committee for Animal Welfare (OPBA) of the Department of Pharmacy, Health and Nutrition Sciences of the University of Calabria (Italy) and in compliance with the Council Directive (86/609/EEC) and Legislative Decree 26/2014 in order to apply the principles of reduction and refinement. The experiments were conducted on Wistar rats (250–300 g), obtained from Charles River Laboratories (Lecco, Italy) and they were housed in transparent polyethylene cages measuring 36 cm × 18.5 cm × 24 cm in a room with controlled temperature (22 ± 1 °C) and with a light-dark program (lights on from 7:00 to 19:00) for at least 7 days before being used. Food and water were available. Rats were sacrificed by exposure to 4% isoflurane followed by cervical dislocation.

### 2.4. Antimicrobial Test

*Escherichia coli* (ATCC 25922) were provided by Remel Microbiology (Thermo Fisher, Waltham, MA, USA). Cells were grown in Muller Hinton Broth (MHB, Difco, Detroit, MI, USA) containing 2 g/L beef infusion solids, 17.5 g/L hydrolyzed casein, 1.5 g/L starch. The final pH was adjusted to 7.4.

### 2.5. Synthesis of D-Mannose Carboxylate

The formation of the D-Mannose carboxylate was performed in order to ensure the subsequent binding between the D-Mannose and the resveratrol [18,19,20,21]. In a 1 L three-necked flask, equipped with a dropping funnel, refrigerant, and magnetic stirrer, filtered flamed under a current of nitrogen, 80 mL of 85% phosphoric acid (H_3_PO_4_) and 2 g of D-Mannose (1.1 × 10^−2^ mol) were added. After 90 min, the solution was kept under rigorous stirring for 5 min, at which time one third of the total amount (6 g) of sodium nitrite (NaNO_2_, 2 g) was added and a change in appearance and color of the solution was observed, which has become frothy yellow; the reaction was left to stand for 100 min during which the solution from yellow turned green until it darkened (this color change always occurred after the addition of NaNO_2_). After the necessary time, a further 2 g of NaNO_2_ were added under stirring and continued stirring for 5 min; thereafter the reaction was allowed to stand for 45 min. The third part of NaNO_2_ (2 g) was then added by repeating the same procedure. After 45 min, 85% formic acid (HCO_2_H, 20 mL) was added to neutralize the excess sodium nitrite; 160 mL of acetone and 400 mL of ethyl ether were cooled separately for 20–30 min and subsequently added to the reaction flask (exothermic reaction), under stirring, in which a color change is visible as the solution has become white. The solvents were removed under reduced pressure, ethanol was added with the consequent formation of a white precipitate which was filtered and dried. The final product, the brown-looking D-Mannose carboxylate (7.68 g) was characterized by FT-IR, UV-Vis and ^1^H-NMR spectrophotometry.

#### Determination of the Content of Carboxylic Groups of D-Mannose Carboxylate

The analytical mixture was prepared by suspending 0.05 g of D-mannose carboxylate in a mixture of 2.5 mL of borate buffer (pH 8.5) and 2.5 mL of aqueous solution of methylene blue (300 mg/L) in a 50 mL flask under stirring and at room temperature for 1 h. The suspension was filtered, and 1 mL was taken to which 8ml of distilled and acidified water with 1ml of 1N HCL was added [18]. This solution was analyzed by UV-Vis spectrometry through a calibration line relative to methylene blue (ε = 86,126 mol^−1^ L). The method is based on the concentration in solution. The resulting amount of unabsorbed methylene blue was calculated and used in the following formula to obtain the content of the carboxyl groups:

(1) $$\frac{n^{\circ }\mathrm{moles}\;\mathrm{COOH}}{\;g\;\mathrm{dry}\;\mathrm{sample}\;}=\frac{\left(7.5-A\right)\times 0.0013}{E}$$

where A represents the absorbance (total amount of free methylene blue in milligrams), E indicates the dry sample weight in grams and L is the wavelength at 664 nm.

The procedure was carried out by inserting separately distilled water for the blank and the acidified solution of methylene blue diluted half with distilled water from which the absorbance and content of the carboxylic groups was obtained in two 4 mL cuvettes.

### 2.6. Esterification of Resveratrol with Methacrylic Acid

The reaction was carried out in accordance with the Steglich esterification procedure. In a 500 mL three-necked flask, equipped with reflux condenser and magnetic stirrer, carefully flamed, and maintained in an inert atmosphere, 100 mL of dry chloroform, 1 g of resveratrol (4.38 × 10^−3^ mol, moles referred to), 0.90 g of DCC and 26.7 mg of DMAP (2.9 × 10^−4^ mol, twentieth part of the moles of resveratrol) were added. After 15 min, 0.369 mL of methacrylic acid was added. The reaction mixture was kept at room temperature and under stirring for 12 h.

The reaction was constantly monitored by TLC (eluent mixture chloroform: methanol 9:1) and then filtered. The solvents were removed under reduced pressure to obtain a yellow powder which was subsequently recrystallized using hot chloroform to eliminate the dicyclohexylurea (DCU) and dried by means of the mechanical pump to obtain 0.7 g of resveratrol methacrylate characterized with FT-IR and ^1^H-NMR.

### 2.7. Condensation Reaction by Esterification between D-Mannose Carboxylate and Resveratrol Methacrylate

As with the esterification reaction between resveratrol and methacrylic acid, the condensation between D-mannose carboxylate and resveratrol methacrylate was performed by the Steglich esterification reaction. In a 100 mL three-necked flask, equipped with a flamed refrigerant and magnetic stirrer and kept in an inert atmosphere, 50 mL of chloroform and 0.33 g D-mannose carboxylate were added. The resulted mixture was kept under stirring until their complete dissolution; next, 0.525 g of DCC (2.5 × 10^−3^ mol) and 0.015 g DMAP (8.2 × 10^−3^ mol, the twentieth part of the moles of resveratrol methacrylate) were subsequently added and the mixture stirred for 15 min. After the necessary time, 0.5 g of resveratrol methacrylate (1.7 × 10^−3^ mol, moles referred to for the calculations of the other reagents) was added. The reaction was mixed at room temperature, under stirring for 24 h heated by an oil bath to a temperature of 60 °C and monitored by TLC (the eluent chloroform: methanol 9:1). The mixture was filtered, and the solvents were removed under reduced pressure. Next, the solid residue was washed with hot methanol in order to remove the dicyclohexylurea waste product (DCU) and the ester obtained from D-mannose carboxylate and resveratrol methacrylate was recovered in amount equal to 0.543 g and characterized by FT-IR, light scattering, and ^1^H-NMR.

### 2.8. Preparation of Microspheres Based on D-Mannose Carboxylate and Resveratrol Methacrylate

In a thermostatic bath at a temperature of 40 °C it was immersed in a glass reactor (100–150 mL), equipped with a mechanical stirrer, dropping funnel and screw cap with pierceable rubber septum, previously flamed in a current of nitrogen and cooled. The polymerization reaction was carried out in accordance with the procedure reported in the literature [22,23,24,25]: 20 mL of n-hexane and 18 mL of chloroform (dispersing phase) were introduced into the reactor and the dispersing phase was stirred mechanically for 30 min. A predetermined quantity of D-Mannose carboxylate-resvetrol methacrylate (0.2 g, 2.41 × 10^−4^ mol) were suitably solubilized in 3 mL of distilled water, sonicating for a few minutes, in order to facilitate the dissolution process, and added to the reaction under nitrogen current. Next, 0.019 g of EBA (1.20 × 10^−4^ mol) was added followed by 1.07 g of ammonium persulfate ((NH_4_)_2_S_2_O_8_, 5 × 10^−3^ mol), used as radical initiator. A suspension has formed in the reactor. The density of the organic phase was adjusted by adding one of the two solvents until an aqueous phase in equilibrium with the organic phase was obtained. 150 µL of sorbitan monooleate (Span 80) were added under nitrogen, after 10 min 150 µL of polyoxyethylene sorbitan monooleate (Tween 80) and after another 10 min 150 µL of tetramethylethylenediamine (TMEDA). The system is kept under stirring for 1 h and at a temperature of 40 °C. The reactor content was filtered in order to recover the microspheres that were then dried (0.121 g) and observed under the microscope and analyzed by FT-IR and light scattering.

#### 2.8.1. Characterization of the Microspheres

The obtained samples were characterized by IR spectrophotometry, dimensional analysis, optical microscopy and swelling degree measurement in aqueous solutions at different pH values (1.2, 6.8 and 7.4).

#### 2.8.2. Dimensional Analysis

The size of the microspheres was determined by dimensional analysis, using a 90 plus particle size analyzer, at 25 °C. The polydipersion index (PI) was also determined [26]. The microspheres were also characterized by Transmission Electron Microscopy (TEM). The suspension of microspheres was stratified onto a carbon-coated copper grid and left to adhere on the carbon substrate for about 1 min. The excess was removed by a piece of filter paper. A drop of 2% phosphotungstic acid solution was stratified and, again, the solution in excess was removed by a tip of filter paper. The sample was air-dried and observed.

### 2.9. Swelling Studies

To evaluate the hydrophilic affinity of the microparticles towards the aqueous environment, their degree of swelling (WR%) was determined. Known aliquots of dry material (10 mg) were placed in glass filters (porosity G2/3) previously wetted, centrifuged (5 min at 2000 g/min) and then weighed. Next, the samples were placed in contact with a solution of potassium chloride-hydrochloric acid (KCl, HCl) buffer at pH 1.2 (to mimic the acidic environment of the stomach), with a buffer of citric acid and disodium hydrogen (Na_2_HPO_4_) at pH 6.8 (in order to simulate the physiological environment of the intestine) and with a buffer of sodium phosphate (Na_2_HPO_4_) at pH 7.4 (with the aim to mimic the conditions of the colon) at 37 °C until the swelling equilibrium is reached.

At predetermined time intervals (1 h, 3 h, 6 h, 24 h) the excess water was removed from the filters by percolation at atmospheric pressure. Subsequently the filters were centrifuged at 3500 rpm for 15 min and then weighed.

The weights recorded at the times listed above were used to calculate the degree of swelling using the equation:

(2) $$\alpha \;\%=\frac{\left(\mathrm{Ws}-\mathrm{Wd}\right)}{\mathrm{Wd}}\;\cdot 100$$

where Ws and Wd represent the respective weights of the swollen and dried microspheres [27].

### 2.10. Impregnation of the Microspheres with Ciprofloxacin

50 mg of microspheres were placed in contact with a solution of ciprofloxacin (10 mg) in 8 mL of ethanol and 2 mL of distilled water. The impregnation was carried out under stirring at room temperature for 3 days, during which the substance solution was absorbed by the matrix with consequent swelling of the mass itself [28]. Finally, the solvent was released by filtration and the obtained microparticles (14 mg) were dried to constant weight. The analysis of the filtration waters, using a UV spectrophotometer, made it possible to calculate the percentage of ciprofloxacin adsorbed by the matrices (LE%) using the following equation:

(3) $$\mathrm{LE}\;\%=\frac{\left(\mathrm{Ai}-\mathrm{Af}\right)}{\mathrm{Ai}}\;\cdot 100$$

where Ai represents the drug concentration in the solution before loading and Af the drug concentration in the solution after its loading.

### 2.11. Release Studies

The release profile of the bioactive substance from the matrix was evaluated by placing an aliquot of impregnated material (2 mg) in 1.3 mL of a buffer solution of potassium chloride-hydrochloric acid at pH = 1.2, an aliquot of impregnated material (2 mg) in 1.3 mL of a buffer solution of citric acid and disodium hydrogen phosphate at pH = 6.8 and another aliquot of impregnated material (2 mg) in a buffer solution of phosphate buffer at pH = 7.4 all within 3 glass filters (porosity G2). The filter was kept stirred at a temperature of 37 °C. At predetermined time intervals, a certain volume was taken from the release solutions and analyzed by UV-Vis spectrophotometry using a calibration diagram (ε = 0.1734 (mL/mg) cm^−1^ and λ = 280 nm). The percentage of substance released was expressed in relation to absorbance. The test was completed within 72 h [29].

### 2.12. Evaluation of the Antioxidant Capacity

Antioxidant activity was tested in rat liver microsomal membranes. These membranes are made up of phospholipids with a high content of polyunsaturated fatty acids and represent the ideal substrate for the lipid peroxidation process. During this reaction the fatty acids are transformed into toxic metabolites such as aldehydes. Malondialdehyde is generated in a constant way and represents a good indicator of the rate of peroxidation [30,31,32,33]. To mimic the lipid oxidation process, a pro-oxidant agent was used, such as *tert*-butylhydroperoxide (*t*-BOOH), which catalyzes the formation of hydroxyl radicals (OH), responsible for peroxidation [27,28,29,30].

#### Formation of Malondialdehyde

1 mL of microsomal suspension (0.5 mg of protein) was added to a solution consisting of 3 mL of 0.5% trichloroacetic acid (TCA), 0.5 mL of thiobarbituric acid (TBA) and 0.07 mL of 0.2% hydroxytoluene butylate (BHT) in 95% ethanol. The samples obtained were incubated in a bath at 80 °C for 30 min and subsequently centrifuged. After incubation, the TBA-MDA complex (pink chromogen) was detected by UV-Vis spectrophotometry at 535 nm. Results were expressed as nmol of MDA/mg of lipid sample protein [27,28,29,30].

### 2.13. Determination of the Minimum Inhibitory Concentration (MIC)

The MIC of the antibacterial compounds was determined by the broth dilution method [34,35]. Briefly, a solution of each compound was diluted, in series, with Muller Hinton Broth (MHB). Next, suspensions of the microorganisms, prepared from overnight cultures, at a concentration of 10^6^ cfu/mL, were added to each dilution in a 1:1 ratio. The growth (or lack of) of the organisms was visually determined after incubation for 24 h at 37 °C. Ciprofloxacin and empty microspheres were used as a control. The lowest concentration in which there was no visible growth (turbidity) was considered as the MIC.

### 2.14. Statistical Analysis

Data were represented as mean ± SD. All experiments were conducted at least three times independently, with 3 or more technical replicates for each experimental condition tested (unless stated otherwise, e.g., when representative data is shown). Statistically significant differences were determined using the Student’s *t*-test or the analysis of variance (ANOVA) test. For the comparison among multiple groups, one-way ANOVA was used to determine statistical significance. *p* < 0.05 was considered significant.

## 3. Results

### 3.1. Synthesis of Methacrylate Ester

**Figure 2:**
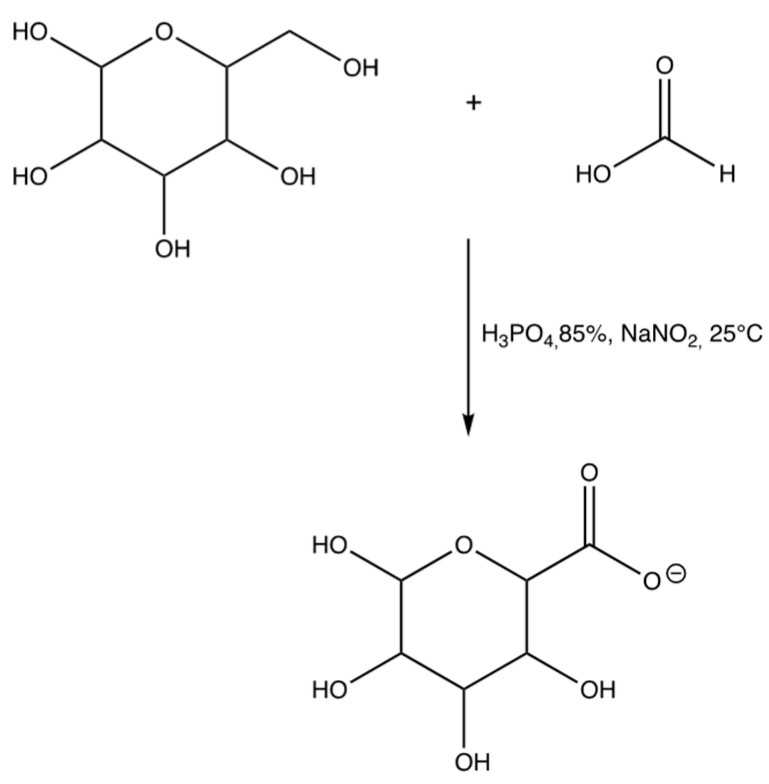
Synthesis of D-mannose carboxylate.

The formation of D-mannose carboxylate was performed to ensure the subsequent binding between D-mannose and resveratrol. The carbonyl oxygen atom of formic acid was protonated by phosphoric acid, in this way the carbonyl carbon was further positively polarized and suffered a nucleophilic attack by the hydroxyl oxygen of D-Mannose, with the formation of a tetrahedral intermediate. A displacement of a proton from alcoholic hydroxyl (D-mannose) to acid hydroxyl (formic acid) was then obtained, allowing the elimination of a water molecule (a better outgoing group than a hydroxyl) and the formation of a protonated ester on the carbonyl oxygen. Finally, deprotonation of the ester took place, with the formation of the final product D-Mannose carboxylate and reformation of the acid catalyst (Figure 2). Quantitative analysis of carboxyl groups [18] found a content of 0.409 g per 0.05 g of D-Mannose carboxylate used. Finally, the product was characterized by FT-IR and 1H-NMR spectrophotometry. FT-IR (KBr) λ (cm^−1^): 3521, 3450 (OH), 2926, 2851 (CH aliphatic), 1725 (C=O), 1383 (OH), 1166 (OH). ^1^H-NMR (D2O): 3.26–3.18 (m, 2H), 3.82 (dd, 2H), 4.0 (d, 1H), 4.8 (d, 1H).

**Figure 3:**
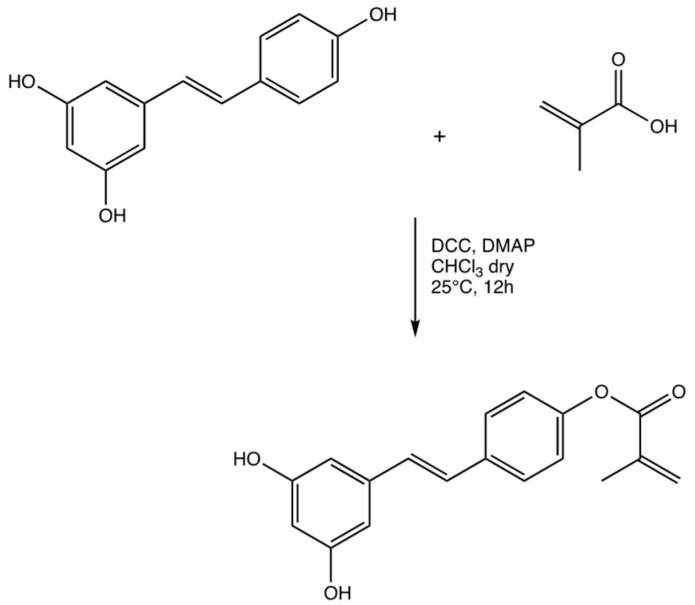
Synthesis of resveratrol methacrylate.

Resveratrol methacrylate was obtained by esterification of Steglich with the carboxylic group of methacrylic acid and the hydroxyl group at the C-1 position of resveratrol. The reaction took place thanks to the help of a condensing agent, DCC, which by binding to the −OH group of methacrylic acid increased the electrophilicity of the carbonyl group favoring the nucleophilic attack by the hydroxyl group in the C-1 position of resveratrol. The use of DMAP acts as both a base and a catalyst: as a basis because it neutralizes any decreases in pH due to the formation of acids and as a nucleophilic catalyst because it removes hydrogen from the hydroxyl group from resveratrol forming the alcoholate that turns out to be more nucleophilic and reactive against the carbonyl carbon of methacrylic acid (Figure 3). Dicyclohexylurea (DCU), a waste product, was removed by hot methanol filtration (scheme 2). Yield 80%. FT-IR (KBr) λ (cm^−1^): 3325 (OH), 3073, 3033 (CH aromatic), 2931, 2853 (CH aliphatic), 1711 (C=O), 1374 (OH), 948, 906 (C=C). ^1^H-NMR (DMSO-d6): 7.29–7.16 (m, 6H), 6.86 (dd, 2H), 6.23 (dd, 1H), 6.25 (d, 1H), 5.73 (d, 1H), 1.9 (s, 3H).

**Figure 4:**
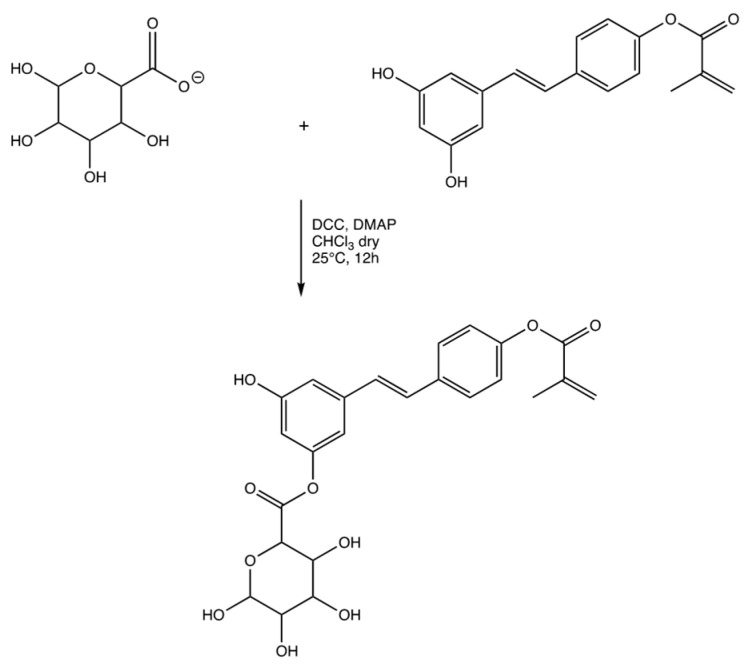
Synthesis of the ester between resveratrol methacrylate and D-mannose carboxylate.

The same process took place in the formation of the final product through the reaction of resveratrol methacrylate and D-Mannose carboxylate (Figure 4). Characterization was carried out by FT-IR and ^1^H-NMR spectrometry (scheme 3). Yield 65%. FT-IR (KBr) λ (cm^−1^): 3502, 3468, 3324 (OH), 3117, 3090 (aromatic CH), 2928, 2852 (aliphatic CH), 1738 (C=O), 1090 (OH), 988, 952 (C=C). ^1^H-NMR (DMSO-d6): 7.65–7.1 (m, 6H), 7.0–6.8 (m, 2H), 6.4 (m, 1H), (6.11 (d, 1H), 5.6 (d, 1H), 4.85 (d, 1H), 4.1 (m, 1H), 3.75 (dd, 1H), 3.2–3.3 (m, 2H) 1.9 (s, 3H).

### 3.2. Preparation of Microspheres Based on D-Mannose Carboxylate and Resveratrol Methacrylate

The microspheres were obtained by reverse-phase emulsion polymerization with an excess of organic solvents not miscible with water (dispersing phase) and an aqueous solution of a monomer (dispersed phase). The agitation ensured the formation of small, dispersed phase drops that took on a spherical shape to decrease their interfacial free energy. Radical polymerization [19] consists of a chain reaction that begins with the formation of free radicals following the cleavage of an appropriate initiator. Radicals react with a monomer molecule by breaking the double bond and simultaneously hydrating another radical. In this case the formed radicals reacted with the methacrylic function present on resveratrol, thus determining the progress of cross-linking. The dispersal phase consists of a mixture of two organic solvents: chloroform (18 mL) and n-hexane (20 mL) while the dispersed phase consists of an aqueous solution (3 mL) containing resveratrol methacrylate-D-mannose carboxylate. By adding one of the two solvents, the density of the organic phase was adjusted to obtain an indifferent equilibrium with the aqueous fase. After 30 min of N_2_ bubbling, the mixture was treated with distilled water containing EBA (comonomer) and ammonium persulfate used as radical initiator. The suspension was subjected to vigorous agitation (900–1000 rpm) to reduce collisions between the drops to counteract their aggregations. These drops are more stabilized in the organic phase thanks to two surfactants: Span 80 and Tween 80. The decomposition of the initiator was accelerated by the addition of TMEDA after 10 min of agitation. The advantage of radical polymerization [19] in reverse suspension is that in the microspheres obtained the formed materials have been solidly cross-linked thanks to the covalent bonds that have been established between the carbon atoms. Chains therefore have greater stability than systems where the same chains have been connected through hydrolysable bonds and/or weak interactions. The average diameter of the particles obtained with this type of polymerization and is between 1 mm^−10^ μm although the most common is between 30–300 μm. It can be said that the average diameter is directly proportional to the interfacial tension between the two liquid phases and to the fraction of the volume of the dispersed phase, while it is inversely proportional to the density of the monomer droplets, the size of the stirring blades and the agitation speed. However, other parameters determine the size of the particles such as: the nature of the substances, the relationships established between them and the characteristics of the reactor such as speed, agitation, shape, and size. The formation of the microspheres was confirmed by FT-IR spectrophotometry. The spectrum shows the absence of the bands typical of the double bond of methacrylic groups. Land dimensions observed by light scattering analysis showed an average diameter of about 0.89 μm and a polydispersion of 0.005.

#### 3.2.1. Swelling Studies

The affinity of the microparticles towards the aqueous media was determined by their degree of swelling (α%) by carrying out swelling studies at three different pH (1.2, 6.8 and 7.4) and at regular time intervals (1 h, 3 h, 6 h, 24 h) [25]. The obtained results, shown in Table 1, showed that the microspheres swell well at pH 6.8 and pH 7.4. 

#### 3.2.2. Morphological Analysis

**Figure 5:**
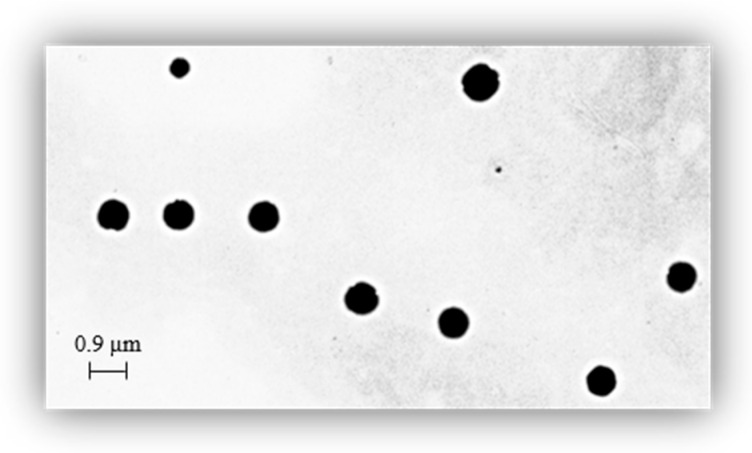
Photomicrographs of microspheres observed by Transmission electron microscopy (TEM).

Morphological analysis of the microspheres, performed by Transmission Electron Microscopy (TEM), confirmed the spherical shape of microspheres (Figure 5).

### 3.3. Impregnation of Microspheres with Ciprofloxacin

In the microspheres the bioactive substance was inserted by means the impregnation technique and when it met the outer surface of the particles established weak interactions with the matrix mostly at the surface level [23,24,25]. The polymer increased in volume in the aqueous solution during impregnation but did not break down in it as it was not soluble while maintaining its three-dimensional structure. The encapsulation efficiency (LE%) of the microspheres was equal to 100%.

### 3.4. Release Studies

**Figure 6:**
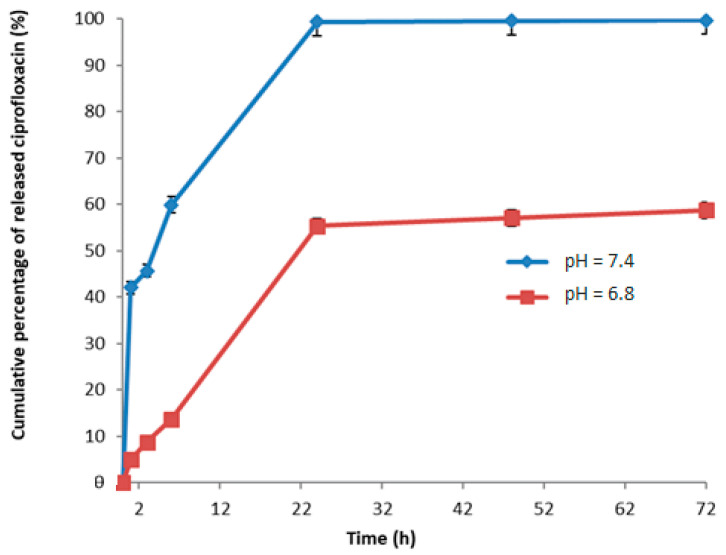
Graph of ciprofloxacin release at pH 6.8 and pH 7.4. The results represent the mean ± SD of three separate experiments. Significance was evaluated by the unpaired *t*-test.

The release studies were carried out on microparticles loaded with ciprofloxacin using two different buffer solutions having 6.8 and 7.4, which are intended to mimic the physiological conditions of intestine at different time intervals (1 h, 3 h, 6 h, 24 h, 48 h, 72 h). The microspheres were kept under stirring at a temperature of 37 °C using a thermostat bath. Spectrophotometric analysis made it possible to evaluate the ability of the polymer matrix to release the drug. The release profile of the biologically active substance was expressed as a percentage of the released substance in relation to the total dose trapped in the matrix, as a function of time (Figure 6).

The data showed that these systems could be used for the site-specific release of ciprofloxacin at pH 6.8 and pH 7.4 (Figure 6). In contrast, negligible release of ciprofloxacin was observed at gastric level (pH 1.2). The amount of ciprofloxacin released by the D-mannose and resveratrol-based microspheres and the trend followed by the releases are not easy to determine and can be influenced by several factors: drug-matrix interactions, chemical-physical nature of the drug loaded into the microspheres, crosslinking of microparticles, penetration rate into the polymer matrix that allows the microspheres to pass from a dehydrated to a swollen state. The volume of cross-linking determines the degree of swelling, i.e., the mechanism of release and the rate of diffusion of the drug from the inside to the outside of the particle. If one of these factors prevails over the others, it can determine the outcome of the release; normally a combination of these factors is established, and the result is obtained. The release can also be influenced by the ability to incorporate the drug into the microspheres, for example, the active ingredient could establish more stable bonds with the microspheres and be released in smaller quantities.

### 3.5. Evaluation of Antioxidant Activity

The antioxidant activity of the microspheres (not containing ciprofloxacin), and of resveratrol, was examined in the microsomal membranes of the Wistar rat liver, in terms of their ability to inhibit the oxidation of lipids, induced by tert-BOOH a generator of free radicals in 120 min of incubation [30].

**Figure 7:**
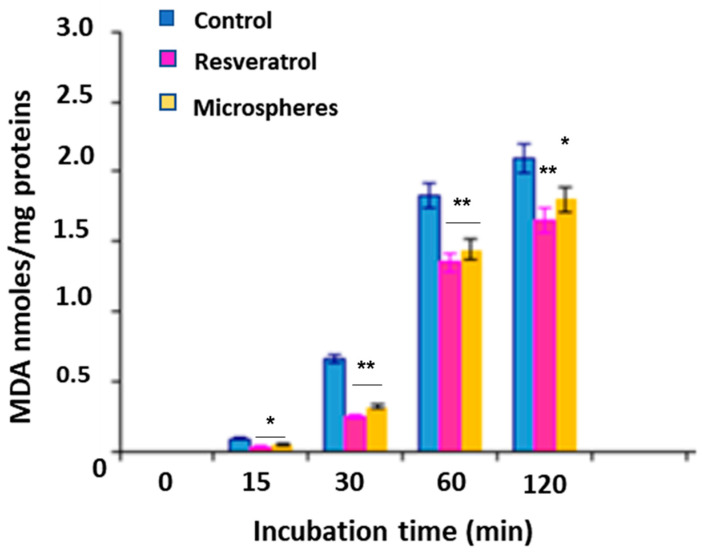
Inhibition of malondialdehyde (MDA) by empty microspheres based on resveratrol and D-mannose. The results represent the mean ± SD of three separate experiments. Significance was evaluated by the unpaired *t*-test. * *p* < 0.05; ** *p* < 0.005.

The results of these tests are shown as a percentage (%) of malondialdehyde inhibition (MDA). The microspheres therefore proved to be powerful antioxidants in a time-dependent manner in protecting microsomal membranes from lipid peroxidation. This result is attributable to the presence in the matrix of resveratrol microspheres, which, despite being methacrylate, still maintained its antioxidant efficacy (Figure 7).

### 3.6. Study of Antimicrobial Activity

**Figure 8:**
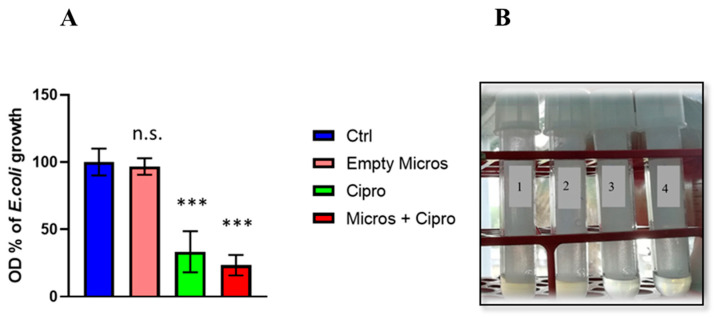
(**A**) Inhibition of bacterial growth after treatment for 24 h with: Ctrl (growth of *Escherichia coli* in the presence of DMSO); Empty Micros (growth of *Escherichia coli* in the presence of empty microspheres); Ciprofloxacin (growth of *Escherichia coli* in the presence of ciprofloxacin 4 μg/mL alone); Micros + Cipro (growth of AND *Escherichia coli* in the presence of microspheres containing ciprofloxacin 4 μg/mL). Data represent the mean fold increase ± SD over Ctrl. One-way ANOVA, Dunnett’s multiple comparisons test n.s. = not significant; *** *p* < 0.0005. (**B**) Representative image of bacterial growth inhibition after 24 h of treatment: 1. Ctrl (growth of *Escherichia coli* in the presence of DMSO); 2. Empty Micros (growth of *Escherichia coli* in the presence of empty microspheres); 3. Ciprofloxacin (growth of *Escherichia coli* in the presence of ciprofloxacin 4 μg/mL alone); 4. Micros + Cipro (growth of *Escherichia coli* in the presence of microspheres containing ciprofloxacin 4 μg/mL).

Ciprofloxacin, empty microspheres and those containing ciprofloxacin were tested at different concentrations (0.5-1-2-4-8 μg/mL). All evaluated samples inhibited bacterial growth (Figure 8A,B). In particular, the best data concerned lee microspheres containing 4 μg/mL of ciprofloxacin [34,35].

## 4. Discussion

In the present work, microspheres based on D-mannose, a natural aldohexose sugar, and resveratrol, a powerful antioxidant, were designed, prepared and characterized.

The preparation of the particles was preceded by the synthesis of the material constituting their matrix. In particular, starting from resveratrol and D-mannose, resveratrol methacrylate and D-mannose carboxylate, respectively, were synthesized and subsequently subjected to a condensation reaction. The formation of the resulting product was confirmed by instrumental analyzes such as FT-IR and 1H-NMR. The microspheres were then made with the product thus obtained by reverse phase emulsion polymerization [23,24,25]. Their characterization by light scattering and electron microscope (TEM) showed spherical and uniform particles. Furthermore, their degree of swelling was evaluated at three different pH values and at defined time intervals. In particular, three buffer solutions were used, one at pH 1.2 to simulate the stomach environment, at pH 6.8 and pH 7.4 to reproduce the conditions of the intestine. The results obtained showed that the microspheres swell well at pH 6.8 and pH 7.4 confirming their possible use as drug delivery systems in the intestinal environment.

The antibiotic ciprofloxacin was then loaded into the microspheres with an encapsulation efficiency of 100%. The data relating to its release from our microspheres are in line with those of other authors [36,37,38]. However, the different trend in terms of release time and quantity of drug released is probably due to the different affinity of ciprofloxacin towards the materials of which the different types of microspheres are made. In our case, the cumulative percentage of drug released, as a function of time, was found to be about 55% at pH 6.8 and 99% at pH 7.4. The latter data is very interesting considering that *E. coli* is usually present in the intestine, so a specific release of ciprofloxacin in this area could inhibit the spread of the bacterium and its subsequent colonization in the urinary tract. Furthermore, studies evaluating the antibacterial activity revealed that the microspheres containing ciprofloxacin (4 µg/mL) were able to inhibit the growth of *Escherichia coli* *E. coli*. Finally, the antioxidant efficacy, evaluated for the presence of derivatized resveratrol in the microsphere’s matrix, and examined in rat liver microsomal membranes in 120 min of incubation [30], confirmed the ability of resveratrol to preserve membranes from oxidation.

## 5. Conclusions

In the present work, microspheres based on D-mannose and resveratrol, have been obtained. They could be potentially useful as carriers for the release of ciprofloxacin in the intestine, and therefore also suitable in the treatment of urinary infections. The idea was, therefore, to exploit both the antioxidant properties of resveratrol and the ability of D-mannose to prevent bacterial adhesion to urothelial cells caused by *E. coli*.

## Figures and Tables

**Table 1:** Swelling degree of microspheres at pH 1.2, pH 6.8 and pH 7.4. The results represent the mean ± SD of three separate experiments. Significance was evaluated by the unpaired *t*-test.

	pH	T (h)			
		1	3	6	24
**α%**	1.2	30 ± 0.9	31 ± 0.9	32 ± 1.0	32 ± 1.0
	6.8	400 ± 12.7	411 ± 13.0	490 ± 15.6	550 ± 17.5
	7.4	487 ± 15.5	500 ± 16.0	744 ± 23.7	765 ± 24.4

## Data Availability

Not applicable.

## References

[1] Dong G., Li J., Chen L., Bi W., Zhang X., Liu H., Zhi X., Zhou T., Cao J. (2019). Effects of sub-minimum inhibitory concentrations of ciprofloxacin on biofilm formation and virulence factors of *Escherichia coli*. Braz. J. Infect. Dis..

[2] Wiles T.J., Kulesus R.R., Mulvey M.A. (2008). Origins and virulence mechanisms of uropathogenic *Escherichia coli*. Exp. Mol. Pathol..

[3] Sanchez C.J., Mende K., Beckius M.L., Akers K.S., Romano D.R., Wenke J.C. (2013). Biofilm formation by clinical isolates and the implications in chronic infections. BMC Infect. Dis..

[4] Donlan R.M., Costerton J.W., Biofilms C.J.W. (2002). Survival mechanisms of clinically relevant microorganisms. Clin. Microbiol. Rev..

[5] Kotian A., Aditya V., Jazeela K., Karunasagar I., Karunasagar I., Deekshit V.K. (2020). Effect of bile on growth and biofilm formation of non-typhoidal salmonella serovars isolated from sea food and poultry. Res. Microbiol..

[6] Hu X., Shi Y., Zhang P., Miao M., Zhang T., Jiang B. (2016). D-Mannose: Properties, production, and applications: An overview. Compr. Rev. Food Sci. Food Saf..

[7] Scribano D., Sarshar M., Prezioso C., Lucarelli M., Angeloni A., Zagaglia C., Palamara A.T., Ambrosi C. (2020). D-Mannose treatment neither affects uropathogenic *Escherichia coli* properties nor induces stable fimh modifications. Molecules.

[8] Pan Y.T., Xu B., Rice K., Smith S., Jackson R., Elbein A.D. (1997). Specificity of the high-mannose recognition site between Enterobacte cloacae pili adhesin and HT-29 cell membranes. Infect. Immun..

[9] Jones C.H., Pinkner J.S., Roth R., Heuser J., Nicholes A.V., Abraham S.N., Hulgretn S.J. (1995). FimH adhesin of type 1 pili isassembled into a fibrillar tip structure in the Enterobacteriaceae. Proc. Natl. Acad. Sci. USA.

[10] Ala-Jaakkola R., Laitila A., Ouwehand A.C., Lehtoranta L. (2022). Role of D-mannose in urinary tract infections—A narrative review. Nutr. J..

[11] Parazzini F., Ricci E., Fedele F., Chiaffarino F., Esposito G., Cipriani S. (2022). Systematic review of the effect of D-mannose with or without other drugs in the treatment of symptoms of urinary tract infections/cystitis (Review). Biomed. Rep..

[12] Lenger S.M., Bradley M.S., Thomas D.A., Bertolet M.H., Lowder J.L., Sutcliffe S. (2020). D-Mannose vs other agents for recurrent urinary tract infection prevention in adult women: A systematic review and meta-analysis. Am. J. Obstet. Gynecol..

[13] Jin-Hyung L., Yong-Guy K., Chaitany J.R., Shi Y.R., Jae J.S., Jintae L. (2019). The anti-biofilm and anti-virulence activities of trans-resveratrol and oxyresveratrol against uropathogenic *Escherichia coli*. Biofouling.

[14] Tian B., Jiayue L. (2020). Resveratrol: A review of plant sources, synthesis, stability, modification, and food application. J. Sci. Food Agric..

[15] Singh D., Mendonsa R., Koli M., Subramanian M., Nayak S.K. (2019). Antibacterial activity of resveratrol structural analogues: A mechanistic evaluation of the structure-activity relationship. Toxicol. Appl. Pharmacol..

[16] Mattio L.M., Dallavalle S., Musso L., Filardi R., Franzetti L., Pellegrino L., D’Incecco P., Mora D., Pinto A., Arioli S. (2019). Antimicrobial activity of resveratrol-derived monomers and dimers against foodborne pathogens. Sci. Rep..

[17] Ratz-Łyko A., Jacek A. (2019). Resveratrol as an active ingredient for cosmetic and dermatological applications: A review. J. Cosmet. Laser. Ther..

[18] Cassano R., Trombino S., Bloise E., Muzzalupo R., Iemma F., Chidichimo G., Picci N. (2007). New BroomFiber (*Spartiumjunceum* L.) Derivates: Preparation and Caracterization. J. Agric. Food Chem..

[19] Cassano R., Di Gioia M.L., Mellace S., Picci N., Trombino S. (2017). Hemostatic gauze based on chitosan and hydroquinone: Preparation, characterization, and blood coagulation evaluation. J. Mater. Sci. Mater. Med..

[20] Trombino S., Curcio F., Poerio T., Pellegrino M., Russo R., Cassano R. (2021). Chitosan Membranes Filled with Cyclosporine A as Possible Devices for Local Administration of Drugs in the Treatment of Breast Cancer. Molecules.

[21] Trombino S., Curcio F., Di Gioia M.L., Armentano B., Poerio T., Cassano R. (2022). Multifunctional Membranes Based on β-Glucans and Chitosan Useful in Wound Treatment. Membranes.

[22] Trombino S., Serini S., Cassano R., Calviello G. (2019). Xanthan gum-based materials for omega-3 PUFA delivery: Preparation, characterization, and antineoplastic activity evaluation. Carbohydr. Polym..

[23] Cassano R., Trombino S., Ferrarelli T., Bilia A.R., Bergonzi M.C., Russo A., De Amicis F., Picci N. (2012). Preparation, characterization and in vitro activities evaluation of curcumin-based microspheres for azathioprine oral delivery. React. Funct. Polym..

[24] Cassano R., Trombino S., Ferrarelli T., Mauro M.V., Giraldi C., Manconi M., Picci N. (2012). Respirable rifampicin-based microspheres containing isoniazid for tuberculosis treatment. J. Biomed. Mater. Res. A.

[25] Trombino S., Cassano R., Mellace S., Picci N., Loizzo M.R., Menichini F., Tundis R. (2016). Novel microspheres based on triterpene saponins from the roots of *Physospermum verticillatum* (Waldst & Kit) (Apiaceae) for the improvement of gemcitabine release. J. Pharm. Pharmacol..

[26] Koppel D.E.J. (1972). Analysis of macromolecular polydispersity in intensity correlation spectroscopy: The method of cumulants. Chem. Phys..

[27] Trombino S., Cassano R., Cilea A., Ferrarelli T., Muzzalupo R., Picci N. (2011). Synthesis of pro-prodrugs L-lysine based for 5-aminosalicylic acid and 6-mercaptopurine colon specific release. Int. J. Pharm..

[28] Iemma F., Spizzirri G., Puoci F., Muzzalupo R., Trombino S., Cassano R., Leta S., Picci N. (2006). pH-Sensitive hydrogels based on bovine serum albumin for oral drug delivery. Int. J. Pharm..

[29] Wolthuis V.D., Franssen O., Talsma H., Kettens-van den Bosch J.J. (1995). Synthesis, Characterization, and Polymerization of Glycidyl Methacrylate Derivatized Dextran. Macromolecules.

[30] Trombino S., Serini S., Di Nicuolo F., Celleno L., Andò S., Picci N., Calviello G., Palozza P. (2004). Antioxidant effect of ferulic acid in isolated membranes and intact cells: Synergistic interactions with α-tocopherol, β-carotene, and ascorbic acid. J. Agric. Food Chem..

[31] Trombino S., Cassano R., Bloise E., Muzzalupo R., Leta S., Puoci F., Picci N. (2007). Design and synthesis of cellulose derivates with antioxidant activity. Macromol. Biosci..

[32] Trombino S., Cassano R., Ferrarelli T., Barone E., Picci N., Mancuso C. (2013). Trans-ferulic acid-based solid lipid nanoparticles and their antioxidant effect in rat brain microsomes. Colloids Surf. B.

[33] Cassano R., Mellace S., Marrelli M., Conforti F., Trombino S. (2017). α-Tocopheryl linolenate solid lipid nanoparticles for the encapsulation, protection, and release of the omega-3 polyunsaturated fatty acid: In vitro anti-melanoma activity evaluation. Colloids Surf. B.

[34] Ozsvari B., Fiorillo M., Bonuccelli G., Cappello A.R., Frattaruolo L., Sotgia F., Trowbridge R., Foster R., Lisanti M.P. (2017). Mitoriboscins: Mitochondrial-based therapeutics targeting cancer stem cells (CSCs), bacteria and pathogenic yeast. Oncotarget.

[35] Parisi O.I., Fiorillo M., Caruso A., Cappello A.R., Saturnino C., Puoci F., Panno A., Dolce V., El-Kashef H., Sinicropi M.S. (2014). Enhanced cellular uptake by “pharmaceutically oriented devices” of new simplified analogs of Linezolid with antimicrobial activity. Int. J. Pharm..

[36] Blandón L.M., Islan G.A., Castro G.R., Noseda M.D., Thomaz-Soccol V., Soccol C.R. (2016). Kefiran-alginate gel microspheres for oral delivery of ciprofloxacin. Colloids Surf B Biointerfaces.

[37] Wu S., Gong Y., Liu S., Pei Y., Luo X. (2021). Functionalized phosphorylated cellulose microspheres: Design, characterization and ciprofloxacin loading and releasing properties. Carbohydr. Polym..

[38] Ravindra S., Varaprasad K., Narayana Reddy N., Vimala K., Mohana Raju K. (2011). Biodegradable Microspheres for Controlled Release of an Antibiotic Ciprofloxacin. J. Polym. Environ..

